# Microbial diversity in camel milk from Xinjiang, China as revealed by metataxonomic analysis

**DOI:** 10.3389/fmicb.2024.1367116

**Published:** 2024-03-11

**Authors:** Miao Sun, Wei Shao, Zhengyu Liu, Xianlan Ma, He Chen, Nan Zheng, Yankun Zhao

**Affiliations:** ^1^Institute of Quality Standards and Testing Technology for Agro-Products, Xinjiang Academy of Agricultural Sciences, Laboratory of Quality and Safety Risk Assessment for Agro-products, Ministry of Agriculture, Urumqi, China; ^2^College of Animal Science Xinjiang Agriculture University, Urumqi, China; ^3^Ministry of Agriculture Laboratory of Quality and Safety Risk Assessment for Dairy Products, Institute of Animal Science, Chinese Academy of Agricultural Sciences, Beijing, China

**Keywords:** camel milk, single molecule real-time, bacterial diversity, psychrophilic bacteria, pathogenic bacteria

## Abstract

The quality of raw camel milk is affected by its bacterial composition and diversity. However, few studies have investigated the bacterial composition and diversity of raw camel milk. In this study, we obtained 20 samples of camel milk during spring and summer in Urumqi and Hami, Xinjiang, China. Single-molecule real-time sequencing technology was used to analyze the bacterial community composition. The results revealed that there were significant seasonal differences in the bacterial composition and diversity of camel milk. Overall, *Epilithonimonas* was the most abundant bacterial genus in our samples. Through the annotated genes inferred by PICRUSt2 were mapped against KEGG database. Non-parametric analysis of the bacterial community prediction function revealed a strong bacterial interdependence with metabolic pathways (81.83%). There were clear regional and seasonal differences in level 3 metabolic pathways such as fat, vitamins, and amino acids in camel milk. In addition, we identified lactic acid bacteria in camel milk with antibacterial and anti-tumor activities. Our findings revealed that camel milk from Xinjiang had serious risk of contamination by psychrophilic and pathogenic bacteria. Our research established a crucial theoretical foundation for ensuring the quality and safety of camel milk, thereby contributing significantly to the robust growth of China’s camel milk industry.

## 1 Introduction

Camel milk, also known as “desert platinum,” is rich in amino acids, vitamins, minerals, and several active ingredients (Benmeziane-Derradji, 2021). Compared with cow milk, camel milk, which is more similar to human milk, contains more than three times the amount of vitamins and a lower content of fat (Yadav et al., 2015). However, the camel milk industry is industrialized and standardized to a low degree. Due to the fact that camels are primarily engaged in free-range grazing, herders typically employ manual milking techniques. One of the biggest challenges facing the development of the camel milk industry is microbial contamination during production, transportation, processing, and storage. In addition, raw camel milk, which is prone to fermentation, is not easy to preserve. This makes raw camel milk more susceptible to various types of bacteria, and eventually spoilage.

High-throughput sequencing has been used to study microbial communities in milk and dairy products. With unprecedented resolution, high-throughput sequencing technology can sequence microbial marker genes, whole genomes, and transcriptomes in almost real time (Imanian et al., 2022). Compared with traditional second-generation sequencing technology, SMRT sequencing technology has strong sensitivity and high accuracy in detecting bacterial 16S rDNA in complex samples. SMRT can locate the annotation results to the species level of bacteria and reveal their potential biological functions, which provide useful information to help identify abnormal changes in or contamination of camel milk (Tang and Liu, 2018). We used PacBio SMRT sequencing technology to sequence the full length of bacterial 16S rDNA and to compare the bacterial diversity of raw camel milk obtained in different sampling seasons (summer and winter) and regions (Inner Urumqi or WLMQ and Hami or HM). Zhang et al. (2020), who applied SMRT sequencing to profile full-length 16S rRNA genes of raw mare milk in Xinjiang, noted that raw mare milk was rich in *Lactobacillus helveticus*, *Lactobacillus plantarum*, *Lactococcus lactis*, and *Lactobacillus kefiranofaciens*. In addition, raw mare milk contained sequences belonging to pathogenic bacteria, such as *Staphylococcus succinus*, *Acinetobacter lwoffii*, *Klebsiella oxytoca*, and *Klebsiella pneumoniae*. Liang and Cao (2022) used SMRT sequencing technology to label the species of common and differential bacteria in raw milk from different provinces in China. In the study, transport distance and transport time positively correlated with the relative abundance of *Pseudomonas weihenstephanensis*.

Psychotropic bacteria utilize nutrients in camel milk to reproduce. These bacteria produce spoilage enzymes that cause the deterioration of camel milk, thereby contributing to significant economic losses. Pathogenic bacteria, including *Listeria*, *Salmonella*, and *Bacillus cereus*, can adversely affect the health of consumers to different extents, sometimes leading to fatal outcomes, and impact the quality of camel milk (Bartula et al., 2023; Bianco et al., 2023). In addition to these spoilage and pathogenic bacteria, several studies have shown that camel milk is rich in probiotics, such as *lactobacillus* (Sharma et al., 2021), *Bacillus subtilis* (Daneshazari et al., 2023), *bifidobacterium* (Yasmin et al., 2020), and *Pediococcus, enterococcus* (Moussaid et al., 2023). These probiotics have a wide range of applications in biotechnology and biomedicine and may be used as microbial cultures in the food system due to their safety and unique functional properties.

The study of the bacterial diversity in cow milk has been comprehensive. China and the European Union have developed relevant quality standards. However, the research on bacterial diversity and contamination sources in camel milk is limited. Several countries have not established specific requirements for microbial quality standards in camel milk. Therefore, the bacterial diversity of camel milk in Xinjiang, China was analyzed in this study. It provides valuable guidance for the safe production of enterprises and the subsequent formulation of microbial standards.

In this study, we sequenced bacterial 16S rDNA using SMRT sequencing technology. The purpose of our study was to investigate and compare the differences in bacterial composition and diversity in camel milk samples from different regions and seasons in Xinjiang and to predict the strains with potential functional characteristics.

## 2 Materials and methods

### 2.1 Sample collection

We obtained 20 camel milk samples from Inner Urumqi (WLMQ) and Hami (HM), Xinjiang, China. The camel milk samples were divided into two categories based on the sampling season: summer (June through August) and winter (February through March) (Table 1). Each camel milk sample was collected from a farm containing more than 50 Bactrian camels. Each sample (500 mL) originated from one camel. The samples were package in sterile sampling bottles and transported at −80°C to the laboratory.

**Table 1:** Detailed information of collected camel milk in Xinjiang, China.

Origin of raw camel milk	Samples sizes	Storage temperature	Sampling seasons and specific quantity
Urumqi	5	−80°C	summer: 5 winter: 5
Hami	5	−80°C	summer: 5 winter: 5

### 2.2 Extraction of metagenomic DNA of bacteria from camel milk

One hundred twenty milliliters of raw camel milk were centrifuged at 13000 r/min at 4°C for 15 min to discard the supernatant, and the precipitate was enriched into a 50 mL centrifuge tube. Total genomic DNA was extracted using the OMEGA Soil DNA kit (M5635-02; Omega Bio-Tek, Norcross, GA, United States), following manufacturer’s instructions, and stored at −20°C for further analysis. The quantity and quality of the extracted DNA were assessed spectrophotometrically (NanoDrop NC2000; Thermo Fisher Scientific, Waltham, MA, United States) and via agarose gel electrophoresis, respectively.

### 2.3 SMRT sequencing of the full length of the 16S rDNA gene

PCR amplification of the nearly full-length bacterial 16S rRNA genes was performed using the forward primer 27F (5’-AGAGTTTGATCMTGGCTCAG-3′) and the reverse primer 1492R (5’-ACCTTGTTACGACTT-3′). The extracted DNA was amplified using two-step PCR. Sample-specific 16-bp barcodes were incorporated into the forward and reverse primers for multiplex sequencing in the second PCR step. Both the two steps of the PCR components contained 5 μL of Q5 reaction buffer (5×), 5 μL of Q5 High-Fidelity GC buffer (5×), 0.25 μL of Q5 High-Fidelity DNA Polymerase (5 U/μL), 2 μL (2.5 mM) of dNTPs, 1 μL (10 μM) of each Forward and Reverse primer, 2 μL of DNA Template, and 8.75 μL of ddH2O. Thermal cycling consisted of initial denaturation at 98°C for 2 min, followed by 25/10 cycles (for first and second amplification step, respectively) consisting of denaturation at 98°C for 30 s, annealing at 55°C for 30 s, and extension at 72°C for 90 s, with a final extension of 5 min at 72°C. A total of PCR amplicons were purified with Agencourt AMPure Beads (Beckman Coulter, Indianapolis, IN) and quantified using the PicoGreen dsDNA assay kit (Invitrogen, Carlsbad, CA, United States). After the individual quantification step, amplicons were pooled in equal amounts, and SMRT technology was performed using the PacBio Sequel platform at Shanghai Personal Biotechnology Co., Ltd. (Shanghai, China).

To decrease the sequencing error rate, PacBio circular consensus sequencing (CCS) reads were derived from multiple alignments of sub-reads. In CCS, DNA polymerase reads a ligated circular DNA template multiple times, which effectively generates a consensus sequence from multiple reads of a single molecule. Raw sequences were initially processed through the PacBio SMRT Link portal (version 5.0.1.9585). Sequences were filtered for a minimum of three passes and a minimum predicted accuracy of 99% (minfullpass = 3, minPredictedAccuacy = 99). The files generated by the PacBio platform were used for amplicon size trimming to remove sequences >2,000 bp.

### 2.4 Bioinformatics analysis of the sequence data

Microbiome bioinformatics were performed using QIIME2 2019.4 (Bolyen et al., 2019) with slight modifications according to the tutorials^1^. Briefly, raw sequence data were demultiplexed using the demux plugin following by primers cutting with cutadapt plugin (Martin, 2011). Sequences were filtered, denoised, and merged. In addition, chimera was removed using the DADA2 plugin to form the amplicon sequence variants (ASV) (Callahan et al., 2016). Non-singleton amplicon sequence variants (ASVs) were aligned with mafft (Katoh et al., 2002) and used to construct a phylogeny with fasttree2 (Price et al., 2009). The raw sequence reads have been deposited in the National Center for Biotechnology Information Short Read Archive under the accession number SRP486654^2^.

Sequence data analyses were mainly performed using QIIME2 and R packages (v3.2.0). ASV-level alpha diversity indices, such as Chao1 richness estimator, observed species, Shannon diversity index, Simpson index, Faith’s PD, Pielou’s evenness, and Good’s coverage were calculated using the ASV table in QIIME2 and visualized as box plots. Beta diversity analysis was performed to assess the structural variation of microbial communities across samples using Bray–Curtis metrics (Bray and Curtis, 1957) and visualized via principal coordinate analysis (PCoA) and unweighted pair-group method with arithmetic means (UPGMA) hierarchical clustering (Ramette, 2007). The significance of differentiation of microbiota structure between groups was assessed by PERMANOVA (permutational multivariate analysis of variance; McArdle and Anderson, 2001). Taxa abundances at the ASV levels were statistically compared among samples or between groups by MetagenomeSeq and visualized as LefSe (linear discriminant analysis effect size) to detect differentially abundant taxa across groups using the default parameters (Segata et al., 2011). Microbial functions were predicted using PICRUSt2 (phylogenetic investigation of communities by reconstruction of unobserved states; Gavin et al., 2019), MetaCyc^3^, and KEGG^4^.

## 3 Results

### 3.1 Sequence abundance and ASV cluster analysis

**Figure 1:**
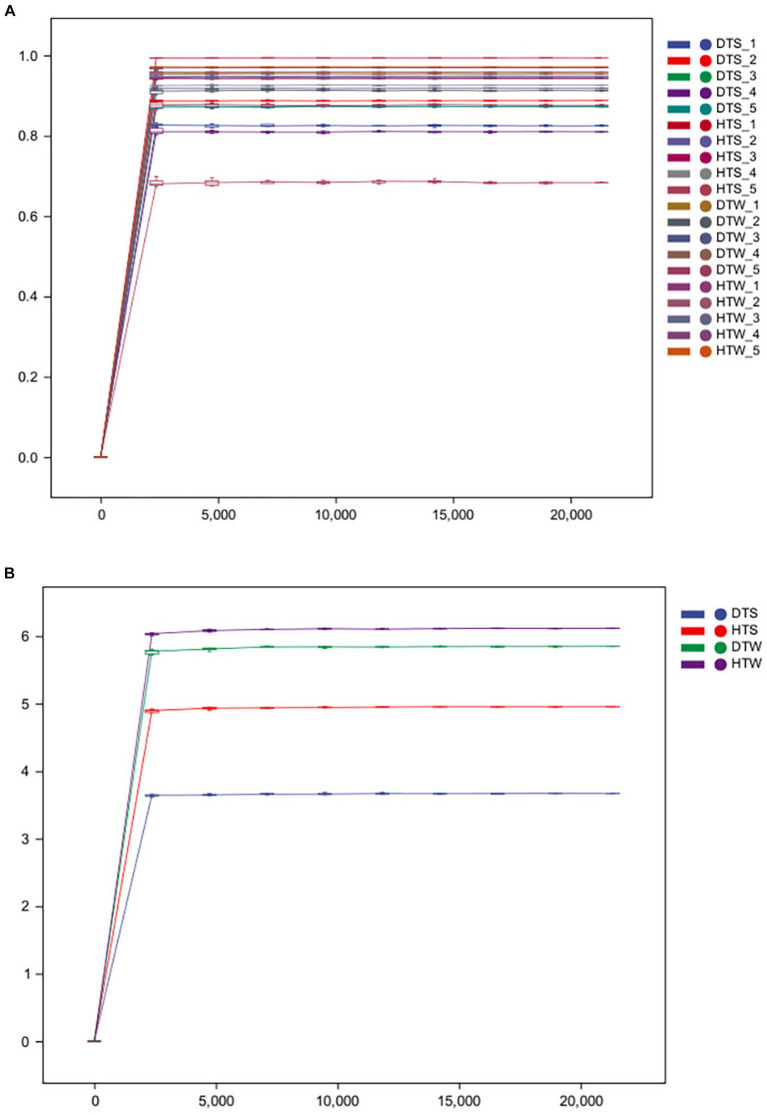
Rarefaction curves of the bacterial communities for each sample **(A)** and each group **(B)** from 20 camel milks. DTS, the sample from Urumqi during summer; DTW, the sample from Urumqi during winter; HTS, the sample from Hami during summer; HTW, the sample from Hami during winter.

After sequencing, all original sequences of the samples were quality-controlled using the DADA2 plug-in in QIIME2 software (Callahan et al., 2016). We obtained 897,481 original 16S rDNA reads. After denoising and dechimerism, 712,675 valid 16S rDNA reads and 672,808 high-quality 16S rDNA reads were obtained. Sparse curves are commonly used in ecology. By randomly drawing a certain number of sequences from each sample, we can predict the species community diversity that the sample is likely to contain at a given sequence depth (Li et al., 2012). Figure 1 shows that, with increasing sequencing depth, the curve of each sample tended to be flat. With increasing sample number, the increase of species was not more obvious, indicating that the current sequencing depth was reasonable and could ensure the accuracy of subsequent analysis.

**Figure 2:**
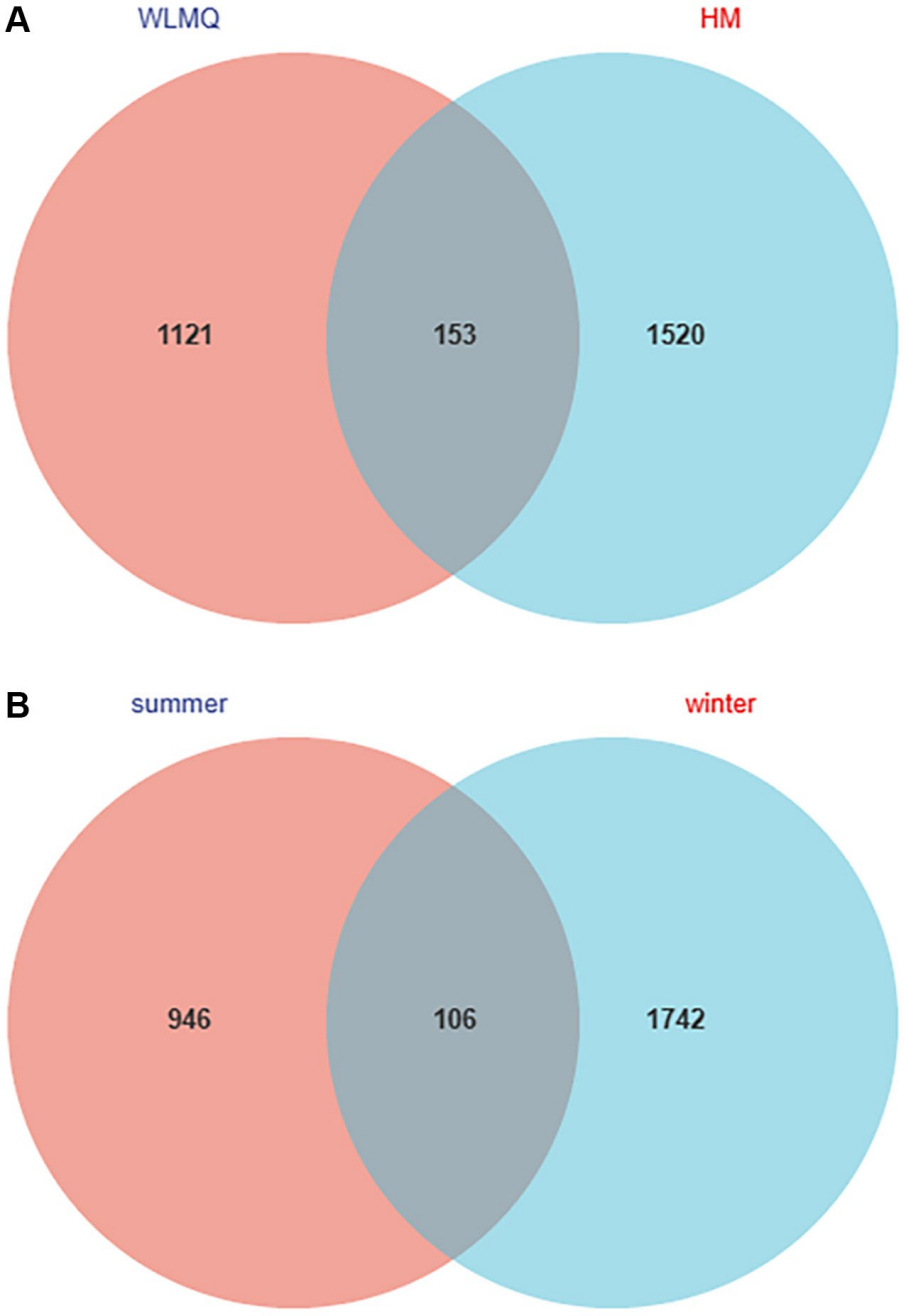
Venn diagram of operational taxonomic units in camel milk from 2 regions in Xinjiang **(A)** and in camel milk obtained in 2 sampling seasons **(B)**. WLMQ, Urumqi; HM, Hami.

The number of shared ASVs for the camel milk samples from two regions was 153. ASVs were 1,673 for HM samples and 1,274 for WLMQ samples. Compared with WLMQ samples, HM samples had a higher number of unique ASVs (1,520), indicating that the bacterial richness of camel milk in HM was higher (Figure 2A).

The number of ASVs in winter and summer were 1,848 and 1,052, respectively. The number of similar ASVs obtained in the two seasons was 106. The unique ASVs were 1,742 for winter samples and 946 for summer samples. Therefore, ASVs were higher for the winter than summer samples (Figure 2B).

### 3.2 Alpha diversity of the microbiome in camel milk

**Figure 3:**
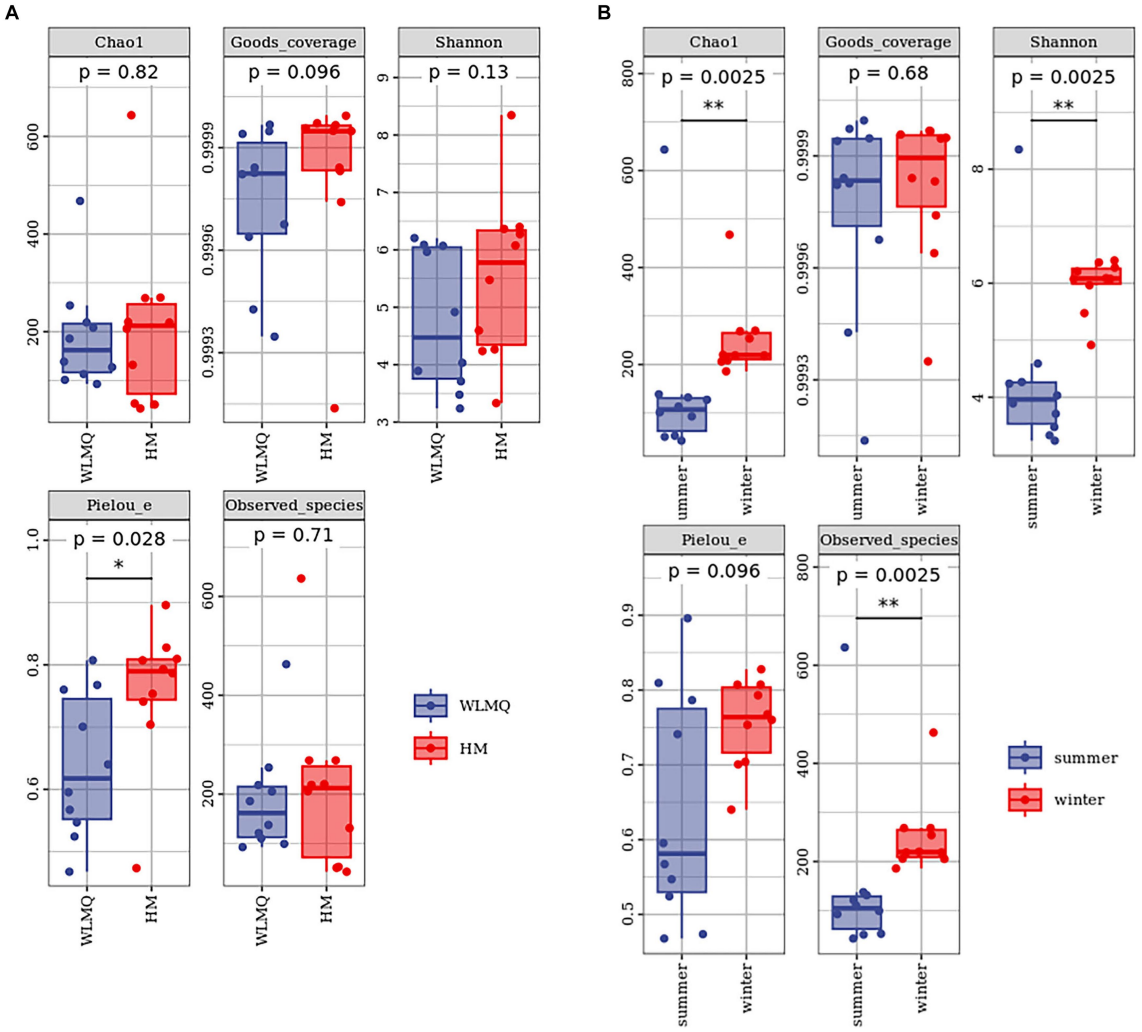
Alpha-diversity index for different groups of 20 camel milk samples. Boxplots show the median (horizontal line), upper and lower quartiles (top and bottom), 1.5× interquartile range (whiskers), and individual data points with dots for the alpha diversity index for different grouped samples. WLMQ, Urumqi; HM, Hami.

Alpha diversity index is a comprehensive index reflecting the richness and evenness of species composition in the sample. The richness of bacterial community was reflected by Chao1 and observed species indices, the diversity was reflected by Shannon and Simpson indices, the uniformity was reflected by Pielou’s evenness index, and the coverage of sequencing samples was reflected by Good’s coverage index. The coverage of each sample was >99%, indicating that the depth of sequencing basically covered all bacteria, and the sequencing results could reflect the real situation of microorganisms in each sample (Table 2). The richness and diversity of bacterial communities were greater in HM samples than in WLMQ samples. Pielou’s evenness index showed that the uniformity of bacterial community distribution was higher in HM samples than in WLMQ samples (*p* = 0.028). The results revealed that the bacterial alpha diversity index of camel milk samples was generally lower in WLMQ than in HM (Figure 3A and Table 2).

**Table 2:** Alpha diversity index of different groups of camel milk samples.

Group	Chao 1	Observed_species	Shannon	Simpson	Pielou_evenness	Good’s coverge
HM	210.58 ± 176.03	209.55 ± 174.13	5.54 ± 1.46	0.93 ± 0.09	0.76 ± 0.11	1 ± 0
WLMQ	190.85 ± 111.63	188.76 ± 111.06	4.76 ± 1.22	0.89 ± 0.05	0.64 ± 0.12	1 ± 0
Summer	149.58 ± 177	147.58 ± 175.12	4.31 ± 1.48	0.87 ± 0.09	0.64 ± 0.15	1 ± 0
Winter	251.86 ± 80.77	250.73 ± 79.5	5.98 ± 0.46	0.94 ± 0.03	0.76 ± 0.06	1 ± 0

According to the sampling seasons, the abundance and diversity of bacterial communities were higher in the winter samples than in the summer samples (Chao 1, *p* = 0.0025; observed species, *p* = 0.0025; Shannon, *p* = 0.0025; Simpson, *p* = 0.013). Pielou’s evenness index showed that the distribution uniformity of bacterial communities in the winter and summer samples was similar (Figure 3B and Table 2).

### 3.3 Bacterial compositions and taxonomic annotation of camel milk

**Figure 4:**
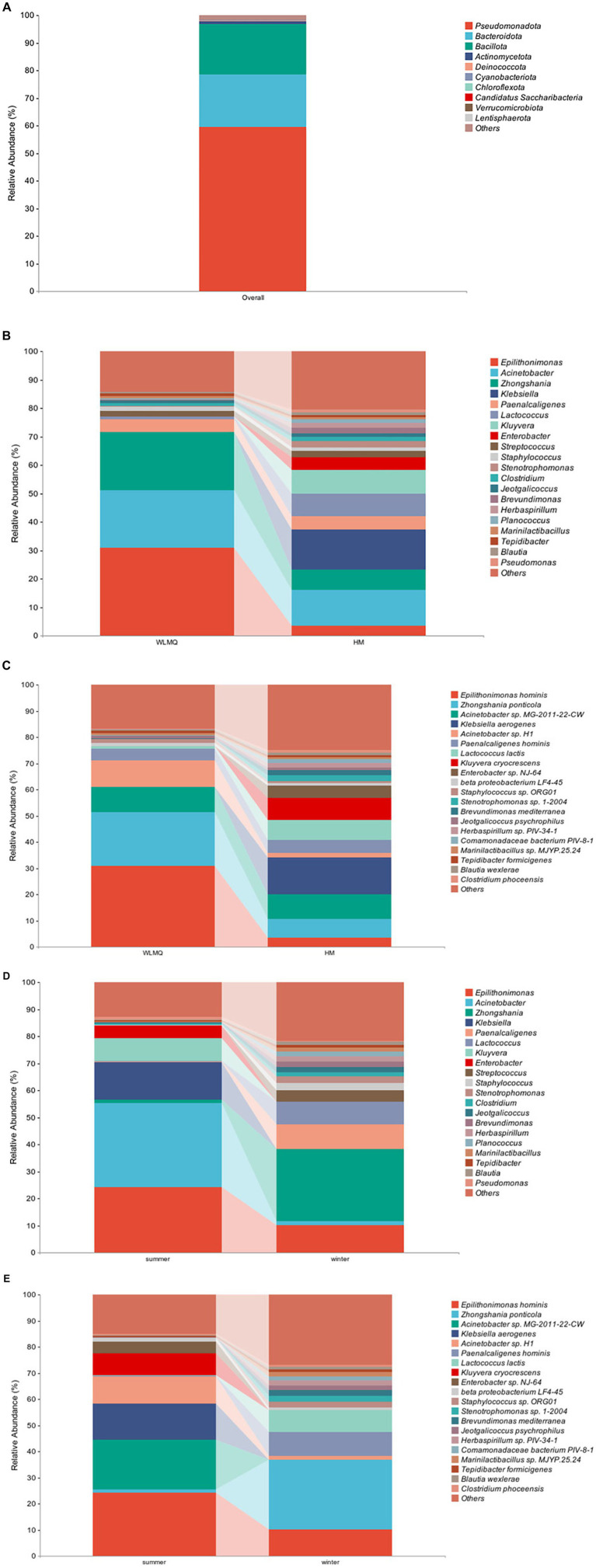
Relative abundance of operational taxonomic units at phylum **(A)** in 20 camel milk. Relative abundance of operational taxonomic units at **(B)** genus and **(C)** species level in camel milk from 2 regions (Urumqi, Hami). Relative abundance of operational taxonomic units at **(D)** genus and **(E)** species level in camel milk obtained in 2 sampling seasons (summer, winter).

To evaluate the bacterial species composition and diversity in camel milk samples, we classified each sample based on six classification levels, including phylum, genus, and species (White et al., 2009). A total of 238 bacterial genera and 532 bacterial species were identified in 20 camel milk samples. The clustering results showed that the bacterial community of all camel milk samples mainly consisted of *Pseudomonadota*, *Bacteroidota*, and *Bacillota* (Figure 4A). At the genus level, the composition and structure of bacterial communities in the samples from different regions were similar, but the proportion of each genus was very different. *Epilithonimonas* was the most abundant genus in WLMQ, while *Klebsiella* was the most abundant genus in HM, accounting for 30.89 and 13.97%, respectively. In addition, we found that *Kluyvera*, while prevalent in WLMQ, was not detected in HM (Figure 4B). At the species level, *Epilithonimonas hominis* was the most abundant species in WLMQ, and *Klebsiella aerogenes* was the most abundant species in HM, accounting for 30.89 and 13.97%, respectively. *Kluyvera cryocrescens* was only identified in HM samples during the summer (8.29%). *K. aerogenes* had the highest relative abundance in HM samples (13.97%), but were negligible in WLMQ samples (Figure 4C).

The bacterial composition of the camel milk samples was different between sampling season and region. According to the results of seasonal group analysis, *Acinetobacter* was the most abundant bacterium in the summer samples (31.09%), but not in the winter samples (1.62%). *Zhongshania* was the most abundant bacterium in winter samples, but not in the summer samples (1.11%). The relative abundance of *Klebsiella*, *Kluyvera*, and *Enterobacter* in the summer samples was 13.98, 8.29, and 4.64% respectively, while none of these bacteria were detected in the winter samples (Figure 4D). At the species level, the composition, but not the proportion, of species types was similar. The bacterial species with the highest relative abundance in the summer samples were *E. hominis*, *Acinetobacter* sp. MG-2011-22-CW, and *K. aerogenes* (24.29, 18.95, and 13.98%, respectively). The bacterial species with the highest relative abundance in the winter samples were *Zhongshania ponticola*, *E. hominis*, and *Paenalcaligenes hominis* (26.65, 10.10, and 9.04%, respectively). The relative abundance of *Z. ponticola* and *P. hominis* was 1.11 and 0.22%, respectively, in the summer samples. Notably, we found that *Acinetobacter* sp. MG-2011-22-CW, *K. aerogenes*, *K. cryocrescens*, and *Enterobacter* sp. NJ-64 were only present in the summer samples (Figure 4E). We inferred that these four strains were tolerant to ambient temperature and that their growth activity was affected by temperature fluctuations (Lechevallier et al., 1988; Behardien, 2008; Jung and Park, 2015).

### 3.4 Beta diversity of the microbiome in camel milk

**Figure 5:**
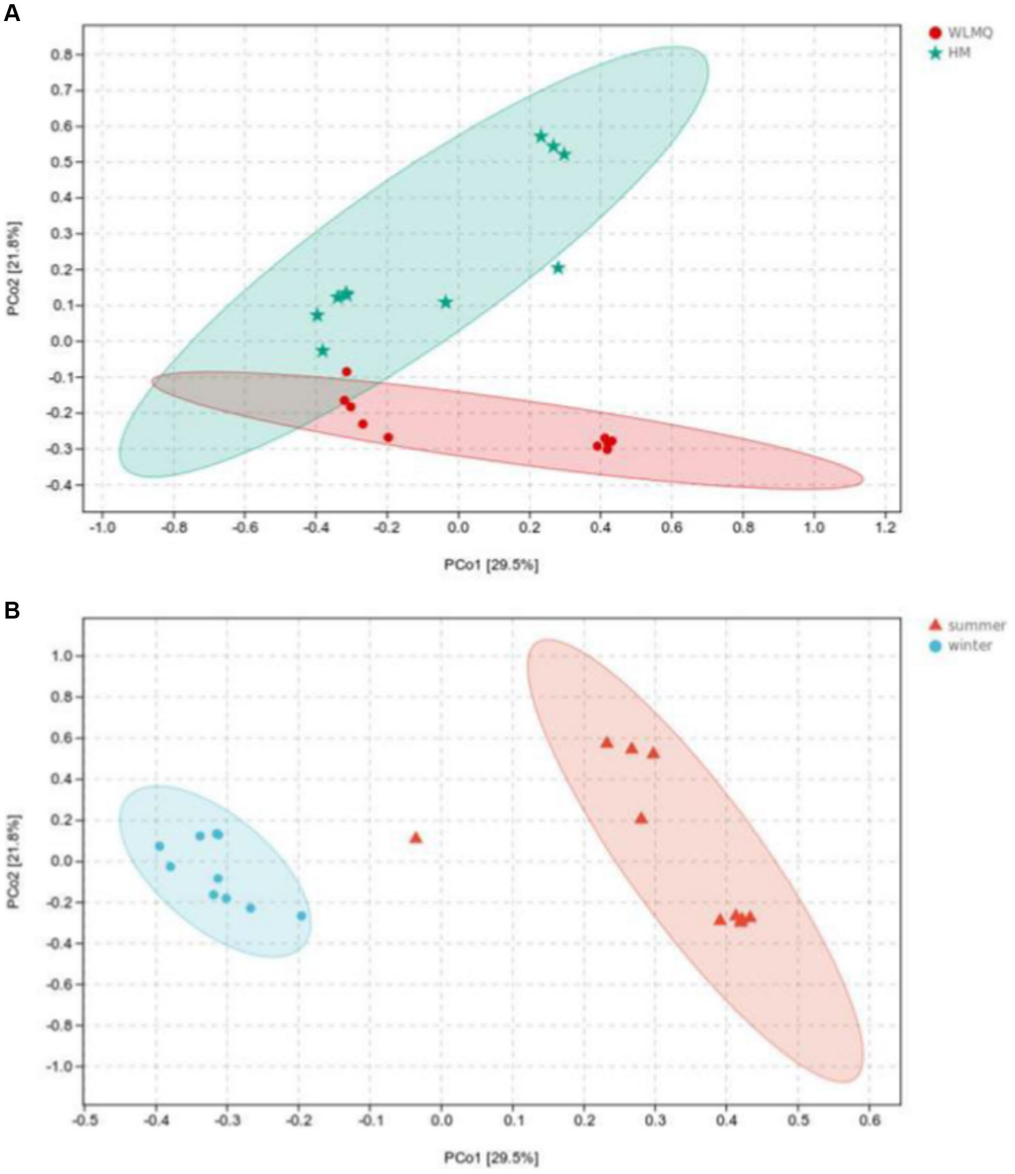
Principal coordinates analysis (PCoA) plots in groups of camel milk from 2 regions **(A)** and camel milk obtained in 2 sampling seasons **(B)** based on Bray–Curtis distance. WLMQ, Urumqi; HM, Hami.

Beta diversity analysis is used to evaluate changes in species composition at spatial and temporal scales. Therefore, the ASV abundance matrix of the samples was analyzed by PCA to study the differences of bacterial community structure in camel milk samples from different regions and seasons. The PCoA generated by beta diversity analysis of the Bray–Curtis distance of the bacteria at the ASV level in raw milk samples from two regions is illustrated in Figure 5A. According to the region grouping, the contribution rates of principal coordinate component 1 (PCoA1) and principal coordinate component 2 (PCoA2) were 29.5 and 21.8%, respectively. The distribution of camel milk samples in the same area showed a certain aggregation, but HTS_5 and DTW_1 samples were scattered. Several camel milk samples from WLMQ were clustered with samples from HM. The analysis results of PERMANOVA showed that the overall difference between different regions was significant (*p* = 0.003). According to the season grouping, the contribution rates of PCoA1 and PCoA2 were 29.5 and 21.8%, respectively. Winter samples showed the most obvious clustering effect, indicating that there was little difference in bacterial communities among winter samples. Summer samples showed a higher degree of dispersion, which suggests that there was a large difference in bacterial communities among summer samples and there was a clearly discrete sample. The analysis results of PERMANOVA showed that there were significant differences in bacterial communities between the seasons (*p* = 0.001; Figure 5B and Table 3).

**Table 3:** Permutational multivariate ANOVA analysis of different grouped camel milk samples based on Bray–Curtis distance.

Characteristic	HM		WLMQ		Summer		Winter	
	Pseudo-F	*p*-value	Pseudo-F	*p*-value	Pseudo-F	*p*-value	Pseudo-F	*p*-value
Region	–	–	–	–	–	–	–	–
HM			4.303	0.003**				
WLMQ	4.303	0.003**	–	–				
Season	–	–	–	–	–	–	–	–
Summer							6.952	0.001**
Winter					6.952	0.001**		

*Difference significant at the 0.05 level and **Difference significant at the 0.01 level.

After obtaining the beta diversity index of the grouped samples, we performed Anosim analysis with the grouped information to assess whether there were significant differences in microbial composition and structure among the sample groups. Statistical analysis was conducted for the accuracy of PCoA analysis. The value of the test statistic R ranged between −1 and 1. When *R* < 0, the difference between groups is greater than the difference between groups. When *R* > 0, the difference between groups is greater than the difference within groups. Based on the grouping of different regions, the difference between the two regions was greater than the difference within the two groups (ANOSIM *R* = 0.300), and the difference between the two groups was significant (*p* = 0.007). When grouped according to the sampling season, the difference between the camel milk samples obtained in the two seasons was greater than the difference within the group (ANOSIM *R* = 0.786), and the difference between the sample groups was significant (*p* = 0.001; Table 4).

**Table 4:** Analysis of similarity (ANOSIM R statistic) of different grouped camel milk samples based on Bray–Curtis distance.

Characteristic	HM		WLMQ		Summer		Winter	
	R	*p*-value	R	*p*-value	R	*p*-value	R	*p*-value
Region	–	–	–	–	–	–	–	–
HM			0.300	0.007**				
WLMQ	0.300	0.007**	–	–				
Season	–	–	–	–	–	–	–	–
Summer							0.786	0.001**
Winter					0.786	0.001**		

*Difference significant at the 0.05 level and **Difference significant at the 0.01 level.

**Figure 6:**
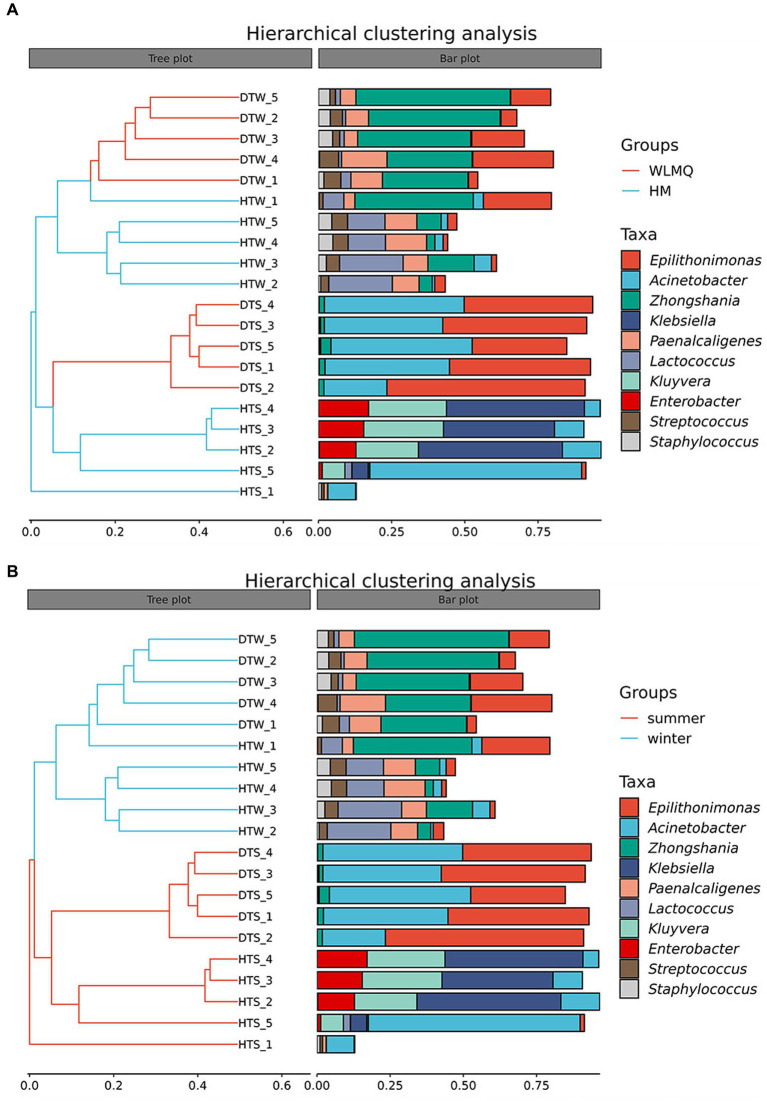
**(A)** Hierarchical cluster analysis in groups of camel milk from 2 region based on Bray–Curtis distance. WLMQ, Urumqi; HM, Hami. **(B)** Hierarchical cluster analysis in groups of camel milk obtained in 2 sampling seasons based on Bray–Curtis distance.

In addition to PCoA analysis, the hierarchical clustering method is used in beta diversity cluster analysis to determine the clustering effect and evaluate the similarity between samples by the branch length of the cluster tree. The results of hierarchical cluster analysis based on Bray–Curtis distance were consistent with those of PCA. As shown in Figure 6, DTW samples clustered with HTW samples in the same branch, and DTS samples clustered with four samples of HTS in the same branch according to different sampling areas. Moreover, winter and summer samples gathered in the upper and lower branches of the clustering tree, respectively, indicating that there were obvious seasonal differences in the bacterial community structure of camel milk samples. In addition, we found that HTW-1 was a single branch in different regions and seasons, with the greatest distance from other samples and the lowest similarity in community structure (Figure 6).

### 3.5 Species difference analysis of bacteria present in camel milk

**Figure 7:**
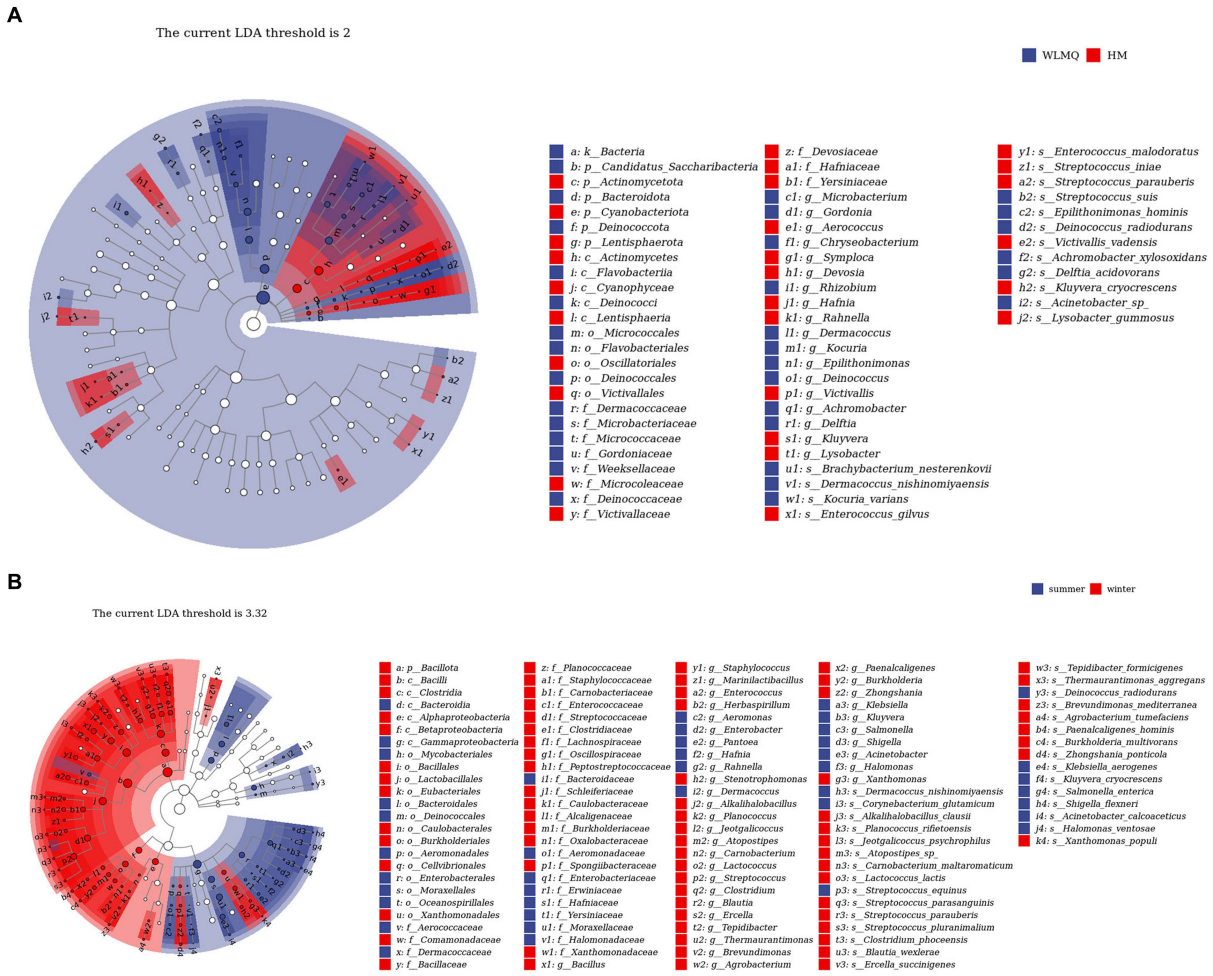
LEfSe analysis identified biomarkers in camel samples from 2 regions **(A)** and camel milk obtained in 2 sampling seasons **(B)**. WLMQ, Urumqi; HM, Hami.

In addition to determining alpha and beta diversity, another major goal in bacterial community structure is to identify indicator communities in camel milk samples from different regions and seasons. We further used LefSe to analyze the bacterial indicator communities of camel milk samples in different groups. The LefSe branch plot shows the enriched groups with LDA scores >2. The higher the LDA score, the more significant the species type. A total of 62 differentially abundant taxon were identified according to different regional groups. Statistically significant biomarkers showed 10 differentially abundant genera and nine differentially abundant species in WLMQ, and eight differentially abundant genera and seven differentially abundant species in HM (Figure 7A). A total of 115 different taxa were identified according to different sampling seasons. Statistically significant biomarkers revealed 12 differentially rich genera and 10 differentially rich species in summer samples and 24 differentially rich genera and 20 differentially rich species in winter samples (Figure 7B).

### 3.6 Functional genes of bacteria present in camel milk

**Figure 8:**
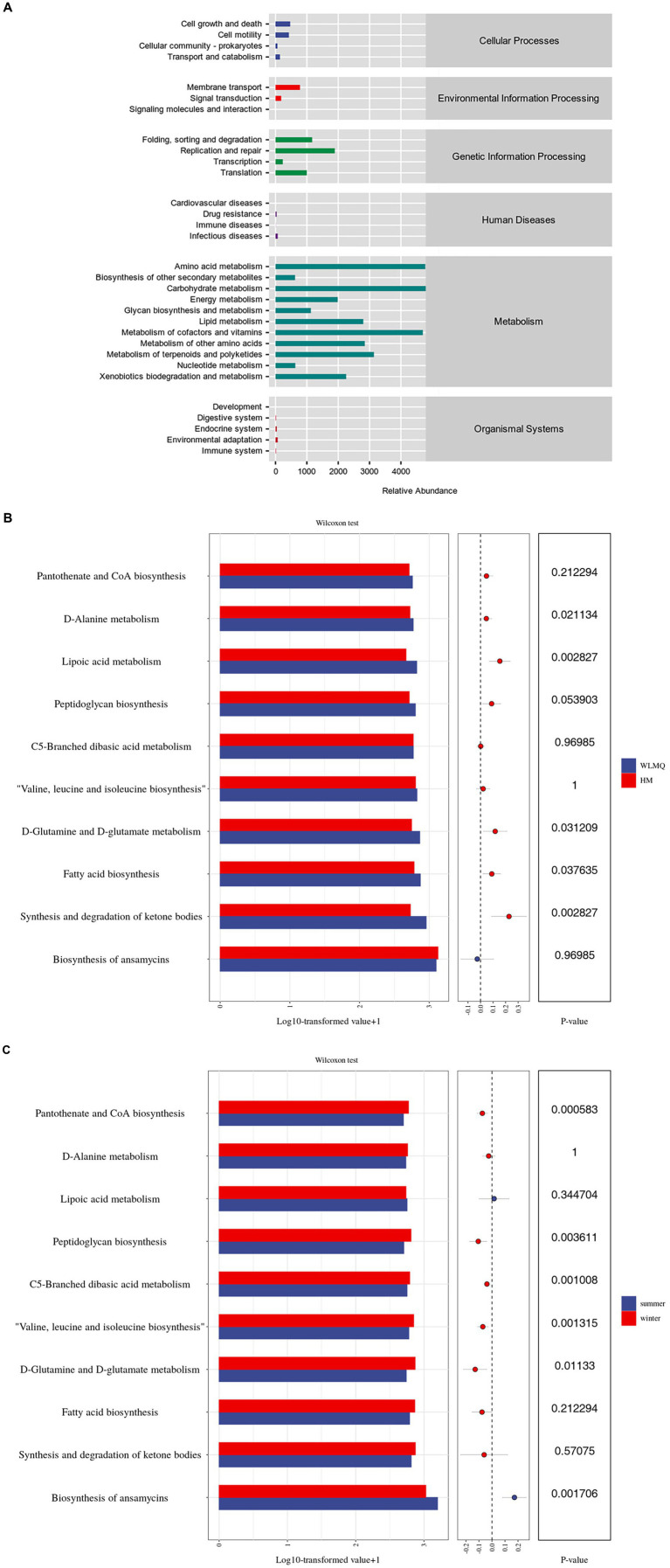
Analysis of the metabolic pathway abundance of camel milk samples based on the KEGG prediction function. The metabolic pathway abundance distribution diagram of all camel milk (level 1 and level 2) **(A)**; pathway level 3 level analysis of metabolic pathway difference of camel milk samples from 2 regions **(B)** and 2 seasons **(C)** (top 10 in relative abundance). WLMQ, Urumqi; HM, Hami.

Based on the 16S rDNA and ASV information of the microbe, we used KEGG and PICRUSt 2 to predict the potential function of the bacterial community in camel milk. A total of six primary metabolic pathways and 31 secondary metabolic pathways were annotated in all samples. The first six metabolic pathways included metabolism (81.83%), genetic information processing (11.82%), cellular processes (3.02%), environmental information processing (2.65%), organismal systems (0.38%), and human diseases (0.30%) (Figure 8A and Supplementary Table S1).

Analysis of sub-pathways of metabolism revealed 31 KEGG pathways in bacterial genes of samples. Amino acid and carbohydrate metabolism were two of the most active level 2 metabolic pathways. Similarly, cofactors, vitamins, and other secondary metabolic pathways were active. Therefore, the function genes related to amino acid and carbohydrate metabolism were generally enriched in camel milk samples. Based on level 3, we further analyzed the metabolic pathways in camel milk in different regions and seasons and observed significant differences in the function of bacterial metabolic genes. Based on the sampling region, further analysis showed that D-alanine, D-glutamine, and D-glutamate metabolism as well as fatty acid biosynthesis were more active in WLMQ samples than in HM samples (*p* < 0.05). The relative abundances of lipoic acid metabolism and of ketone body synthesis and degradation were significantly higher in WLMQ samples than in HM samples (*p* < 0.01) (Figure 8B). Overall, functional genetic traits showed higher significant differences between the two seasons compared to the two regions. Based on the sampling season, peptidoglycan biosynthesis (*p* < 0.01); C5-branched dibasic acid metabolism (*p* < 0.01); valine, leucine, and isoleucine biosynthesis (*p* < 0.01); D-glutamine and D-glutamate metabolism (*p* < 0.05); and pantothenate and CoA biosynthesis (*p* < 0.001) were more active in the winter samples than in the summer samples. Only ansamycin biosynthesis was more active in the summer samples than in the winter samples (*p* < 0.01) (Figure 8C). In conclusion, the relative abundance of metabolism-related genes was the highest among the samples, indicating that metabolism-related genes play important roles in bacterial metabolic function.

## 4 Discussion

In China, camel milk is regarded as a nutritious beverage with a unique nutritional value (Seifu, 2023). However, camel milk represents a natural medium for bacterial growth and thus is susceptible to contamination by pathogenic and spoilage microorganisms. Camels are mainly concentrated in the sparsely populated Gobi desert in Northwest China. Local farmers and herdsmen prefer to consume raw camel milk without boiling it. In addition, several processing industries use low-temperature powder spraying technology to produce camel milk powder. Hence, if the harmful microorganisms in camel milk surpass the prescribed standards, their elimination becomes challenging, thereby contributing to food safety and public health concerns. Elrofaei et al. (2021), who collected 30 raw camel milk samples from Sudan, reported that *Staphylococcus* spp. and *Streptococcus* spp. were the dominant bacteria, with isolation rates of 24.3 and 21.6%, respectively. Serda et al. (2018), who collected 384 camel milk samples, concluded that the average level of *Staphylococcus aureus* was 4.2 × 10^4^ CFU/mL, with an infection rate of 11.45%. These results reveal that raw camel milk is handled under poor sanitary conditions with a high health risk to consumers.

Most countries do not have specific requirements on the psychrophilic or pathogenic bacterial counts that affect the quality and safety of camel milk. Being the leading producer of camel milk, Kenya adheres to the current standard DKS 2061: raw whole camel milk – specification from 2016. According to this standard, the total microbial colony count in raw camel milk should not exceed 2 × 10^6^ CFU/mL, with *Escherichia coli* levels required to be below 1 × 10^5^ CFU/mL. Currently, there are only two local and two industry standards in China that stipulate that the total levels of microbial colonies in raw camel milk should be less than 2 × 10^6^ CFU/mL, but no specifications have been provided for the content of pathogenic bacteria such as *E. coli* and there is no current national standard. Therefore, it is of great significance to study the bacterial composition and diversity in camel milk for the industrialization and standardization of camel milk in Xinjiang, China.

In our study, the SMRT sequencing technology results showed that the bacteria in camel milk were mainly *Pseudomonadota*, *Bacteroidota*, and *Bacillota*. *Epilithonimonas* was the most abundant bacterium in the camel milk samples, followed by *Acinetobacter* and *Zhongshania*, which were also the bacteria genera with the highest relative abundance in WLMQ samples, but accounted for less than 15% in HM samples. Zhao et al. (2020) reported that main bacterial genera in Inner Mongolia camel milk were *Pseudomonas*, *Thermus*, and *Streptococcus*. Wei et al. (2021) found that *Pseudomonas*, *Acinetobacter*, and *Lactobacillus* had high relative abundance in camel milk from China. *Epilithonimonas* spp. is a genus of bacteria that widely exists in the natural environment, such as rivers, lakes, and soil. It is a relatively rare genus of psychrophilic bacteria; researchers have detected the bacteria in raw cow milk and camel milk (Shaked et al., 2010; Quigley et al., 2013). In our study, we found that *Epilithonimonas* was the most abundant genus of bacteria in camel milk in WLMQ samples, and *E. hominis* was the most dominant bacterial species in WLMQ. In our previous field investigation, we observed that the breeding environment of camel farms in WLMQ was poor, which might have contributed to contamination with *Epilithonimonas* through the soil or feces. Moreover, *Epilithonimonas* proliferated and gradually became the dominant bacterial group during the cold chain transport after sample collection. However, its involvement in milk spoilage remains unclear. Therefore, we believe that it is necessary to improve the sanitary conditions during the production process of camel milk to reduce the effect of psychrophilic bacteria on the quality of camel milk. Future studies should evaluate the effect of *E. hominis* on the nutritional composition and quality of camel milk.

*Acinetobacter*, *Klebsiella*, and *Kluyvera* were the dominant bacteria in HM samples. The difference in bacterial composition might be attributed to environmental factors. For example, Urumqi is in the mountainous basin of Xinjiang, while Hami is in the desert. Moreover, Urumqi has a temperate continental climate, while Hami has an arid climate. *Acinetobacter* spp. MG-2011-22-CW, *K. aerogenes*, *K. cryocrescens*, and *Enterobacter* spp. *NJ-64* were detected only in summer samples. *K. aerogenes* and *K. cryocrescens* were found only in the same batch of HM samples. *Klebsiella* is a typical zoonotic pathogen that causes pneumonia, septicemia, urinary tract infections, and soft tissue infections. Donkor et al. (2007), who analyzed 96 cow milk samples from Ghana, reported that 16.7% of the samples contained *Klebsiella*. Yang et al. (2021) reported that 1,063 isolates from 857 cow milk samples from Jiangsu and Shandong, China, contained 104 *Klebsiella* strains with a frequency rate of 9.78%. Jindal et al. (2021), who collected 153 mixed cow milk samples from farms of different sizes, reported that the prevalence of *Klebsiella* was 19.6%. The principal pathogenic reservoirs for transmission of *Klebsiella* are the gastrointestinal tract and the hands of a dairyman. Because of its capacity to spread quickly in the cattle farm environment, *Klebsiella* tends to cause severe diseases (Merhi et al., 2023). *K. aerogenes* is commonly associated with hospital outbreaks and with several infections, including blood infections, skin and tissue infections, respiratory infections, and urinary tract infections. Multidrug resistant strains of *K. aerogenes* have emerged and are responsible for the high mortality rate in ICU patients. *K. aerogenes* has become a major opportunistic pathogen with epidemic potential (Passarelli-Araujo et al., 2018; Umar et al., 2021; Ghafari, 2022). In the past few years, attention has been directed towards sporadic occurrences of *K. aerogenes*, particularly those instances where it harbors class A carbapenemase genes (blaGES-5 and blaKPC-2) and class B carbapenemase genes (blaAIM-1) (Yoshino et al., 2016). In this study, the proportion of *K. aerogenes* in HM samples reached 13.97%, which indicates that it is urgent to solve the contamination of camel milk. We suggest that farms conduct professional and systematic training of milking staff and regularly disinfect milking tools. A recent study found that proper cleaning and disinfection of milking machines can reduce the incidence of *Klebsiella* spp. by 92% (Ágredo-Campos et al., 2023). *Kluyvera* infections in humans are rare and the pathogenic role of *Kluyvera* remains uncertain.

In addition, some pathogens are common bacteria with low relative abundance detected in all camel milk samples, such as *E. coli*, *Pseudomonas aeruginosa*, *B. cereus*, and *S. aureus*. The nutritional value of camel milk lies in its high concentration of natural antibacterial compounds, such as lysozyme, bacteriocin, and lactoferrin. It has been reported that lactic acid bacteria inhibits the growth of pathogenic bacteria by producing organic acids (e.g., lactic acid and acetic acid) and that lactobacillus can adapt well to acidic pH conditions, showing strong antibacterial activity (Muhammad et al., 2014). Lactic acid bacteria are well-known producers of bacteriocins (e.g., nisin, pediocin, and lacticin), which are antimicrobial peptides (Yang et al., 2018; Gao et al., 2019). The research of Benaouda et al. (2023) confirmed that lactic acid bacteria inhibit bacterial growth. We infer that the growth of pathogenic bacteria is inhibited by the antibacterial activity of lactic acid bacteria in camel milk (Mahmoudi et al., 2019; Rahmeh et al., 2019). This is an exciting possibility that could be investigated in future research.

The composition and diversity of bacterial communities vary greatly among different sampling seasons. The PERMANOVA results showed that both sampling season (calculated by Bray–Curtis distance: *p* = 0.001, *R*^2^ = 0.786) and geographic location (Bray–Curtis distance: *p* = 0.007, *R*^2^ = 0.3) were significant factors affecting the bacterial composition of camel milk. However, analyses based on sampling season consistently yielded higher *R*^2^ values compared to sampling region, suggesting that the sampling season was the more deterministic factor in contributing to significant differences in the bacterial composition in camel milk. Our results are consistent with the results of Guo et al. (2021), who reported that the bacterial community of raw milk showed high diversity in terms of seasonal factors and found that the bacterial diversity of raw milk in the winter was higher than in the summer or autumn.

Microorganisms are involved in metabolic reactions such as anabolism and catabolism to obtain energy and nutrients needed for survival and growth. Carbohydrate catabolism is usually the energy source for microbial growth and development during the fermentation process of dairy products, and microorganisms in dairy products metabolize carbohydrates such as lactose through the carbohydrate metabolism pathway (Pan et al., 2014; Rizo et al., 2020). In our study, carbohydrate metabolism was the most active metabolic pathway, and the metabolites produced by this pathway can usually activate the complex enzyme system in the fermentation process of camel milk through the carbon–oxygen transport system of lactic acid bacteria, thereby impacting its acidity, sweetness, and taste.

Lactic acid bacteria inhibit the growth of pathogenic bacteria by metabolizing organic acids. According to the functional prediction, we found that fatty acid biosynthesis was active. We concluded that lactic acid bacteria may inhibit the growth of some pathogens in camel milk by metabolizing fatty acids. Valine, leucine, and isoleucine are essential amino acids that play important roles in protein synthesis, energy metabolism, and muscle growth and repair. Wang et al. (2021) and Peng et al. (2022) have shown that the biosynthesis of valine, leucine, and isoleucine promotes the growth of certain probiotics. In our study, anabolic functions of valine, leucine, and isoleucine contributed to a high relative abundance with *Lactococcus*, indicating that the anabolic function of these three amino acids in camel milk is strong.

We unexpectedly found that ansamycin biosynthesis had the highest functional abundance in our study. The biosynthesis of ansamycins is usually carried out by Gram positive bacteria. Ansamycins are broad-spectrum antibiotics with antibacterial and antitumor activities. They work by inhibiting Hsp90 (Heat Shock Protein 90) in bacteria or tumor cells (Escalante et al., 2021; Skrzypczak and Przybylski, 2022). Bhutia et al. (2021) reported a significant positive correlation between ansamycin biosynthesis and *Clostridium* and *Enterococcus* abundance. In our study, *Clostridium* and *Enterococcus* had a high relative abundance, consistent with the results by Bhutia et al. (2021). In addition, psychrobacter abundance was significantly positively correlated with the synthesis and degradation of ketone bodies (Bhutia et al., 2021). In our study, psychrophilic bacteria are the main dominant bacteria group. Considering that the synthesis and degradation of ketone bodies are important in the process of lipid metabolism (Fukao et al., 2004; Chandel, 2021; Ma et al., 2021), the high abundance of this metabolic function indicates that the psychrophilic bacteria metabolize fat (Jin et al., 2022). We speculate that psychrophilic bacteria in camel milk metabolize proteins, fat, and carbohydrates. The degradation products have a serious impact on the flavor, texture, and nutritional value of camel milk and dairy products (Samaržija et al., 2012; Jin et al., 2022; Qin et al., 2023). We used Wilcoxon test to compare the differences of main metabolic functions in different groups. We found that the camel milk samples obtained in different regions and seasons were susceptible to bacterial action, which impacted the content of fatty acids, vitamins, and amino acids in camel milk. Even though camel milk is rich in beneficial microorganisms, few studies have evaluated them or their related functional genes. This will require us to explore the functional genes of beneficial microorganisms in a more in-depth and targeted way, which is also our future research plan. The results of our study can be used to evaluate the possible sources of bacterial contamination in camel milk, implement real-time and effective prevention and control measures, make breakthroughs in the subsequent processing and safety of camel dairy products, and discover known bacterial species with functional development potential through functional prediction analysis.

## 5 Conclusion

In this study, we used the PacBio SMRT sequencing technology to evaluate and compare the bacterial diversity of raw camel milk obtained in different regions and seasons of Xinjiang. We found that there were significant differences in bacterial diversity and metabolic function in samples from different regions and seasons. *E. hominis* was the most abundant bacterial species in camel milk. Our investigation systematically examined the diversity and gene functions of bacteria in camel milk from Xinjiang. This analysis aims to provide comprehensive microbial background information for subsequent activities in dairy processing, ensuring dairy safety, exploring bacterial species, and potentially identifying probiotics.

## Data availability statement

The original contributions presented in the study are included in the article/Supplementary material, further inquiries can be directed to the corresponding author.

## Author contributions

MS: Writing – original draft, Writing – review & editing, Data curation, Formal analysis, Investigation, Supervision. WS: Writing – original draft, Supervision. ZL: Writing – original draft, Conceptualization, Investigation. XM: Writing – review & editing. HC: Writing – review & editing. NZ: Writing – review & editing. YZ: Writing – review & editing, Funding acquisition, Resources.

## Footnotes

^1^ https://docs.qiime2.org/2019.4/tutorials/

^2^ https://www.ncbi.nlm.nih.gov/sra/?term=SRP486654

^3^ https://metacyc.org/

^4^ https://www.kegg.jp/
