# Supplementary material for: Microbial diversity in camel milk from Xinjiang, China as revealed by metataxonomic analysis
Source: Front Microbiol. 2024 Mar 11;15:1367116. doi: 10.3389/fmicb.2024.1367116 (PMC10964795; doi:10.3389/fmicb.2024.1367116)
Supplement: Supplementary file 1 [file Table_4.DOCX]

| **Supplementary Table 2: Predictive pathways with abundance inferred by PICRUSt2 against KEGG database.** | | | |
| --- | --- | --- | --- |
| **Sl No.** | **Sub-Pathways** | **Abundance** | **%** |
| 1 | Carbohydrate metabolism | 4808.09 | 13.25% |
| 2 | Amino acid metabolism | 4779.15 | 13.17% |
| 3 | Metabolism of cofactors and vitamins | 4699.52 | 12.95% |
| 4 | Metabolism of terpenoids and polyketides | 3141.22 | 8.65% |
| 5 | Metabolism of other amino acids | 2849.92 | 7.85% |
| 6 | Lipid metabolism | 2799.61 | 7.71% |
| 7 | Xenobiotics biodegradation and metabolism | 2254.78 | 6.21% |
| 8 | Energy metabolism | 1984.15 | 5.47% |
| 9 | Replication and repair | 1890.28 | 5.21% |
| 10 | Folding, sorting and degradation | 1169.19 | 3.22% |
| 11 | Glycan biosynthesis and metabolism | 1127.54 | 3.11% |
| 12 | Translation | 998.68 | 2.75% |
| 13 | Membrane transport | 779.97 | 2.15% |
| 14 | Nucleotide metabolism | 631.38 | 1.74% |
| 15 | Biosynthesis of other secondary metabolites | 624.28 | 1.72% |
| 16 | Cell growth and death | 468.14 | 1.29% |
| 17 | Cell motility | 425.93 | 1.17% |
| 18 | Transcription | 232.35 | 0.64% |
| 19 | Signal transduction | 180.69 | 0.50% |
| 20 | Transport and catabolism | 140.43 | 0.39% |
| 21 | Infectious diseases | 71.45 | 0.20% |
| 22 | Environmental adaptation | 69.47 | 0.19% |
| 23 | Cellular community - prokaryotes | 61.77 | 0.17% |
| 24 | Endocrine system | 42.05 | 0.12% |
| 25 | Drug resistance | 34.88 | 0.10% |
| 26 | Digestive system | 13.34 | 0.04% |
| 27 | Immune system | 12.63 | 0.03% |
| 28 | Immune diseases | 3.45 | 0.01% |
| 29 | Cardiovascular diseases | 0.07 | 0.00% |
| 30 | Signaling molecules and interaction | 0.01 | 0.00% |
| 31 | Development | 0 | 0.00% |
