# Supplementary figures and images for: Microbial diversity in camel milk from Xinjiang, China as revealed by metataxonomic analysis
Source: Front Microbiol. 2024 Mar 11;15:1367116. doi: 10.3389/fmicb.2024.1367116 (PMC10964795; doi:10.3389/fmicb.2024.1367116)

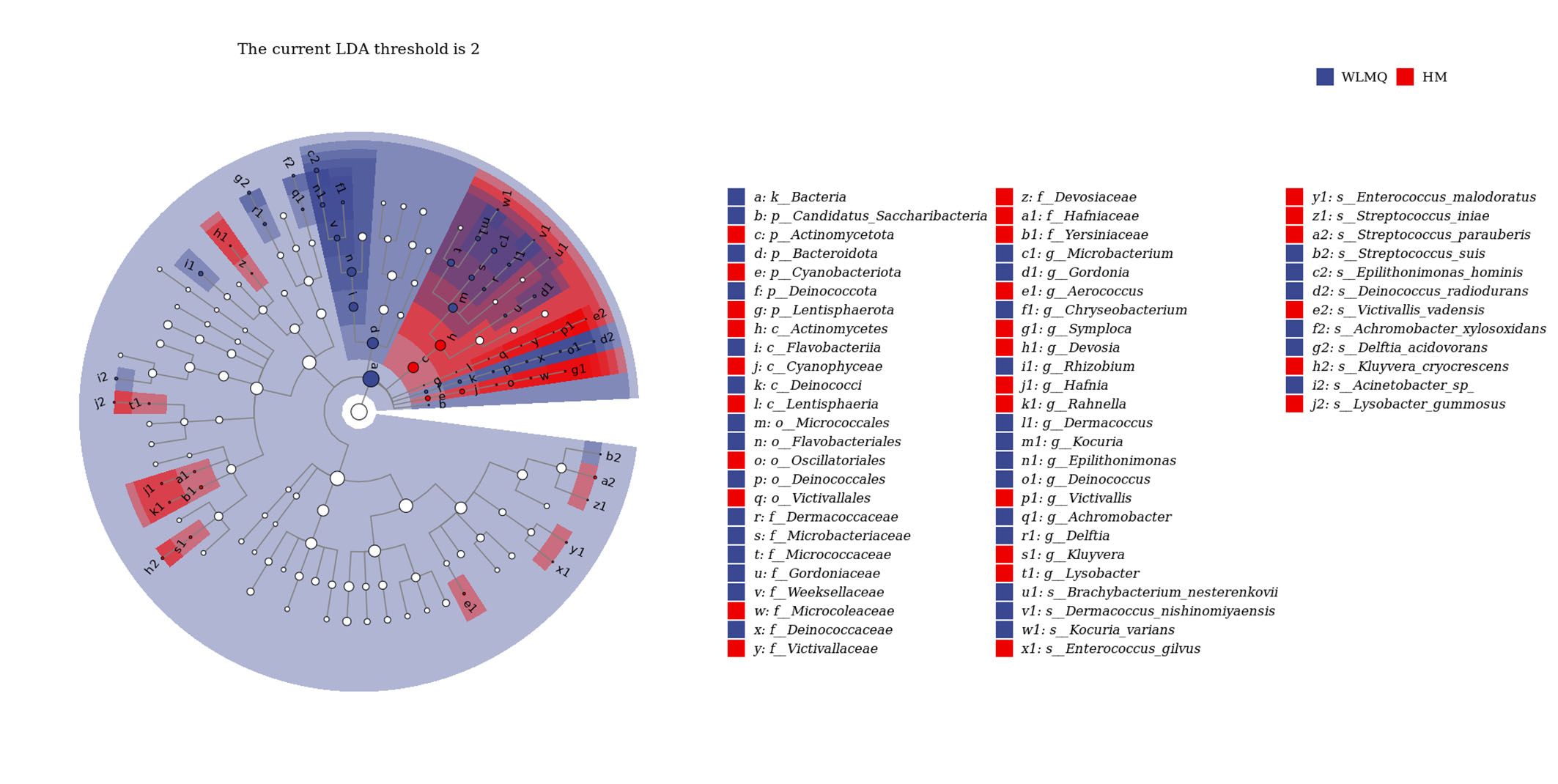

Supplement: Supplementary file 2 [file Image_1.JPEG]

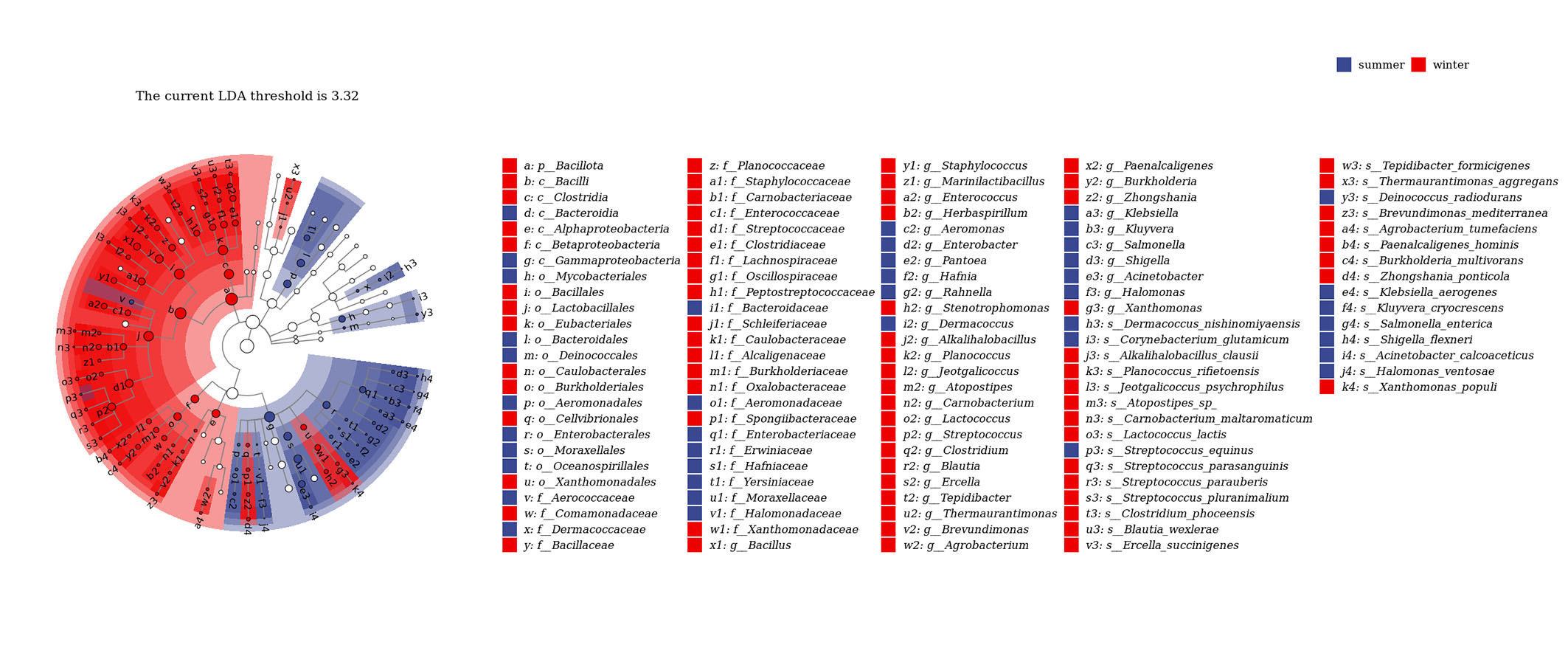

Supplement: Supplementary file 3 [file Image_2.JPEG]

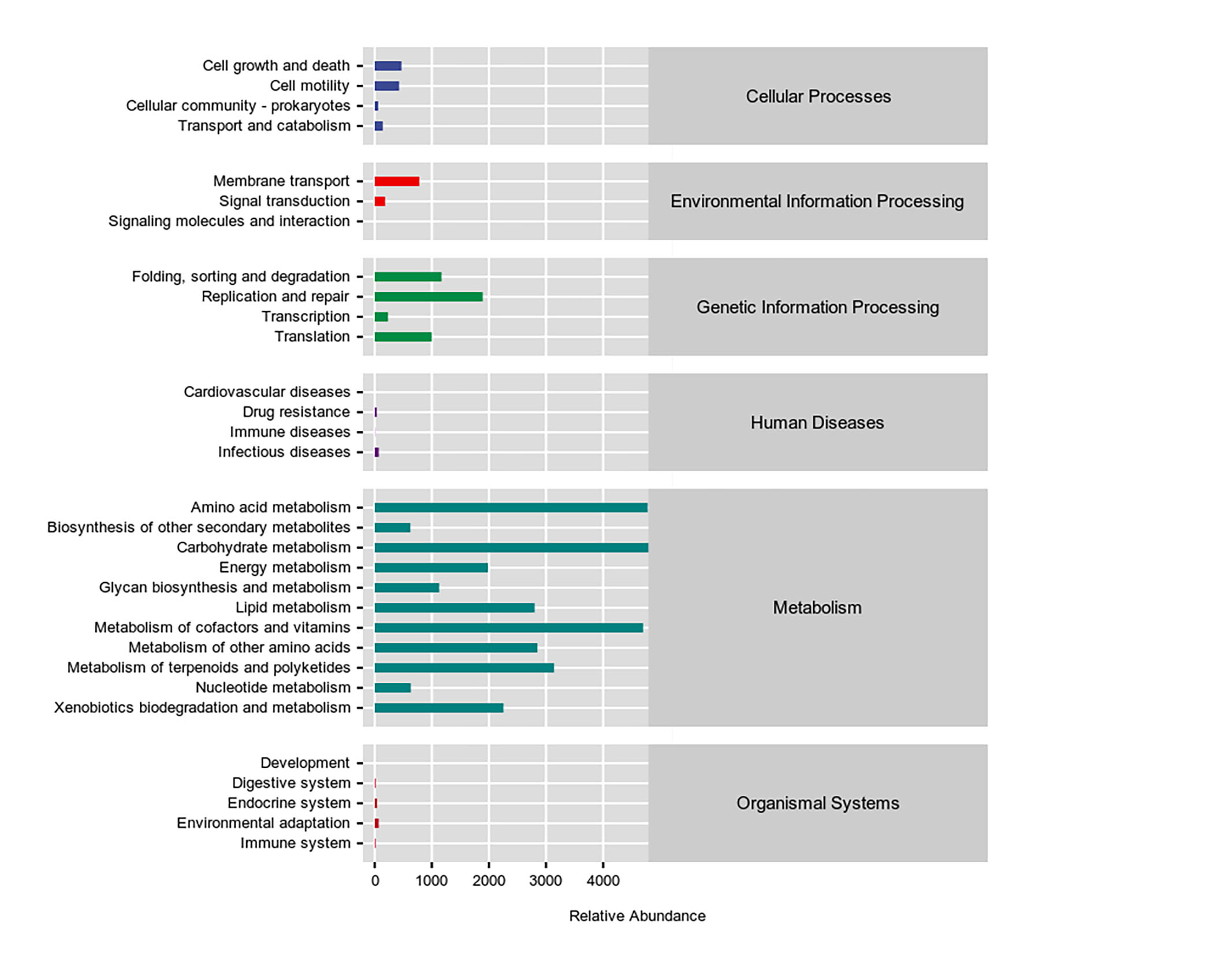

Supplement: Supplementary file 4 [file Image_3.JPEG]

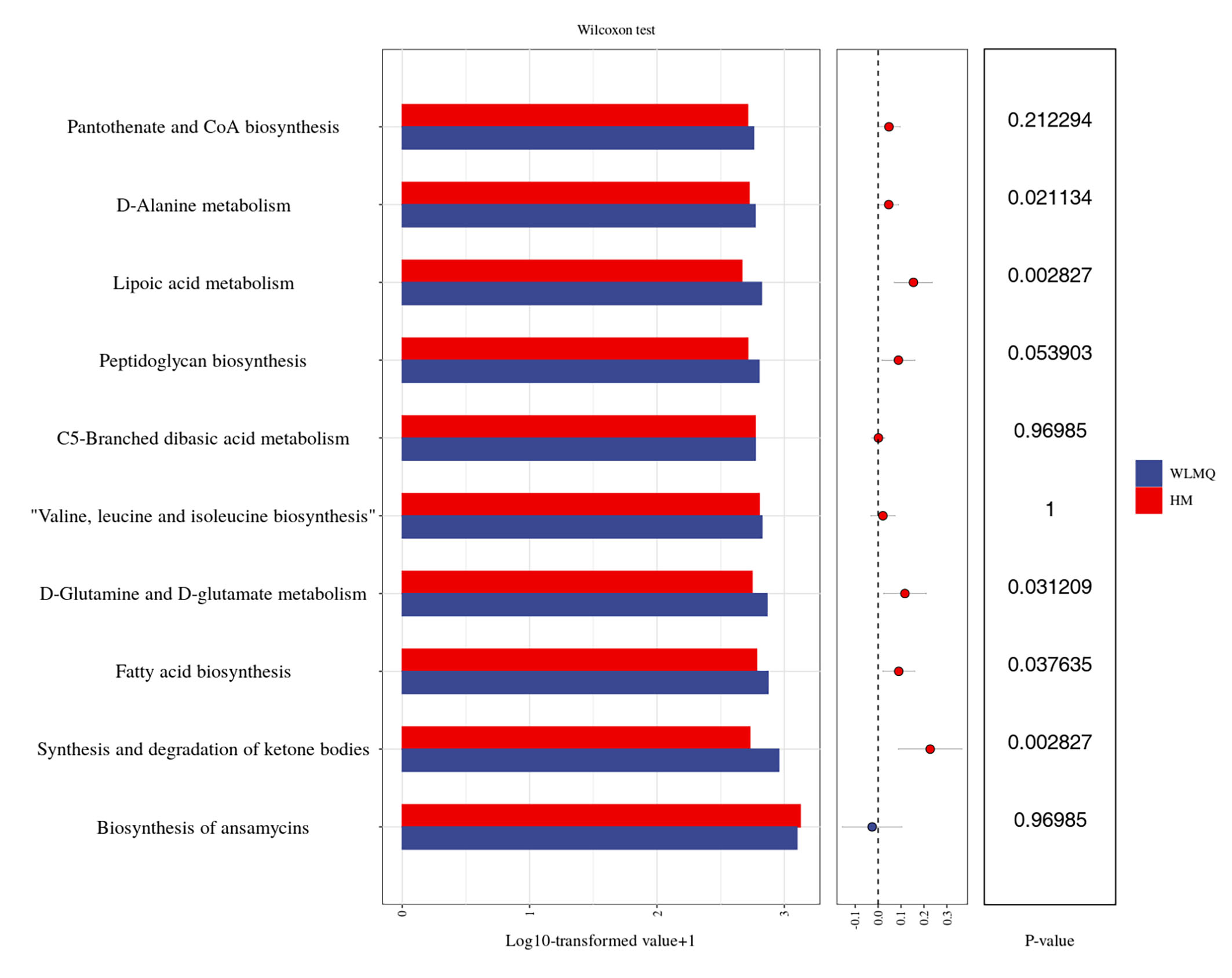

Supplement: Supplementary file 5 [file Image_4.JPEG]

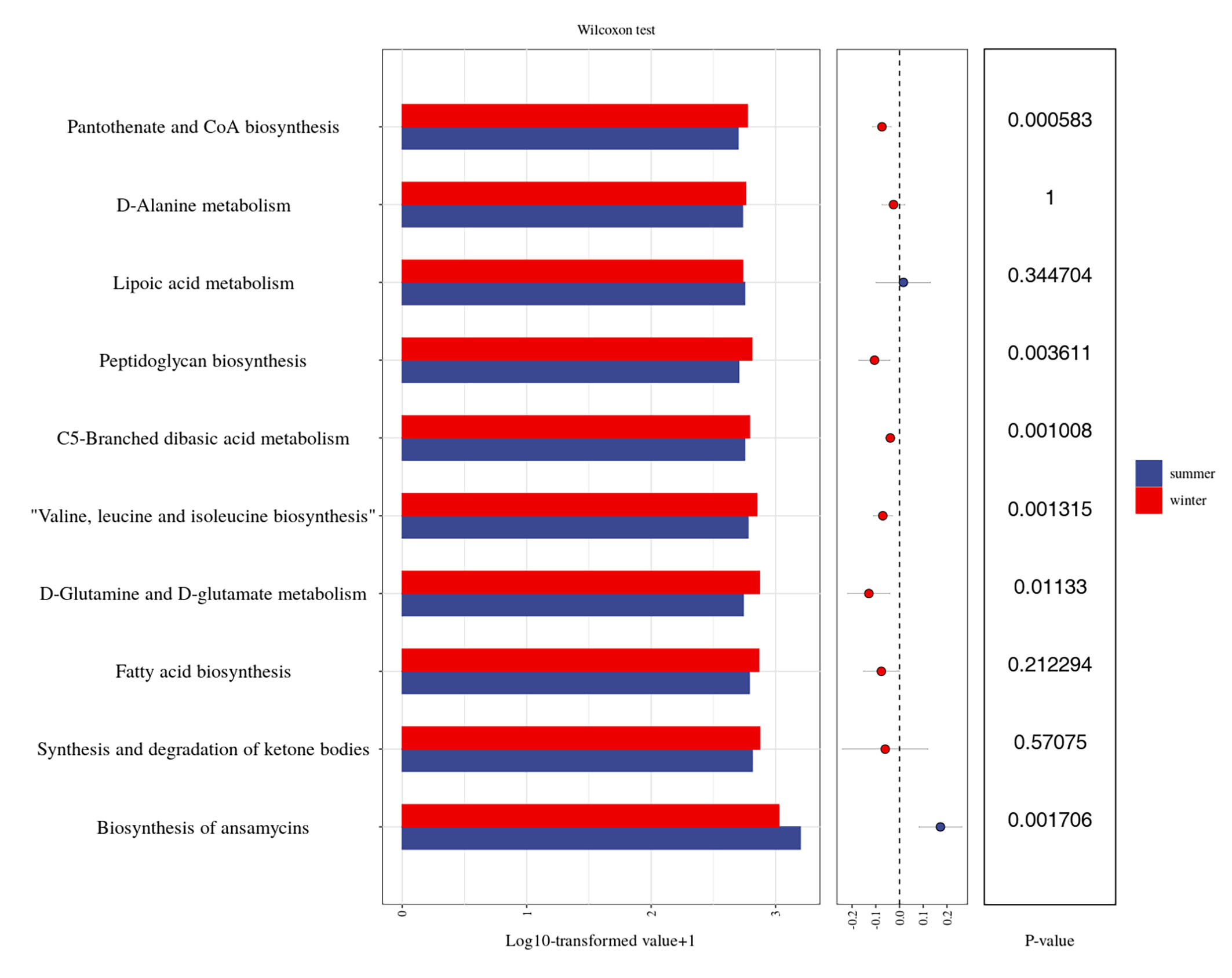

Supplement: Supplementary file 6 [file Image_5.JPEG]
